# Research status of the relationship between microecological imbalance and lung cancer

**DOI:** 10.3389/fmicb.2025.1558379

**Published:** 2025-03-10

**Authors:** Xin Jin, Yangang Gu, Xiaojie Song

**Affiliations:** ^1^Department of Clinical Pharmacy, Qilu Hospital (Qingdao), Cheeloo College of Medicine, Shandong University, Qingdao, China; ^2^Department of Respiratory and Critical Care Medicine, Qilu Hospital (Qingdao), Cheeloo College of Medicine, Shandong University, Qingdao, China

**Keywords:** microecology, microecological imbalance, microbiota, lung cancer, human health

## Abstract

Microecology refers to the ecosystem formed by human and microbial communities in the process of co-evolution, the microecological imbalance is associated with occurrence and development of multiple diseases, including lung cancer. In this review, we detailedly summarized the concept and roles of microecology, the relationship between microecology and human diseases, and related techniques in microecology studies. Importantly, we specially analyzed the correlations between microecology and lung cancer by focusing on gut microbiota, oral microbiota and lower respiratory tract microbiota, and further evaluated the effects of microbiota dysbiosis on chemotherapy and immunotherapy efficacy in lung cancer. At last, we discussed the potential mechanisms by which dysregulated microbiota promotes the genesis and development of lung cancer. Microecology-centered detection and intervention will improve the early diagnosis of lung cancer and provide new targets for the treatment of lung cancer.

## Background

Microorganisms are tiny organisms that cannot be seen with the naked eye but can only be seen with the help of a microscope. These include non-cellular microorganisms such as viruses, prokaryotes such as bacteria, mycoplasma, chlamydia, and eukaryotes such as fungi (Kodio et al., 2020). Berger’s Manual of Systematic Bacteriology divides prokaryotes into archaea and bacteria domains so that bacteria, archaea, and eukarya constitute the biological world’s three domain systems (Boone et al., 2001). Archaea have unique structures and metabolic patterns that can survive in extreme environments, such as methanogens, thermophilic acidophilic bacteria, and unnecessary halophilic bacteria with poor pathogenicity (Krzmarzick et al., 2018). Berger’s Manual of Systematic Bacteriology classifies bacteria and archaea into kingdoms, phyla, classes, orders, families, genera, and species for easy study and application (Boone et al., 2001). With the development of science and technology, the current research on microorganisms is becoming more and more accurate, and many research requirements are to reach the level of strains or even subspecies or types.

The microbiome is a community of microorganisms, including bacteria, fungi, and viruses. Many kinds of microorganisms colonize the body surface and communicate with the outside world or the host across multiple body sites (Cullin et al., 2021). Microecology refers to the ecosystem formed by human and microbial communities in the process of co-evolution, which depend on each other, restrict each other, and coexist peacefully (Lu et al., 2020). For example, intestinal microecology comprises gut microbiota, intestinal epithelial cells and mucosal immune system (Lin et al., 2019). In addition, the skin on the human body, digestive tract, respiratory tract, oral cavity, and urogenital tract all contain rich and unique microecology (Webster, 2007; Cen et al., 2018; Sun et al., 2021; Lin et al., 2024; Wei W. et al., 2022). These microbial communities enter the fetus shortly after birth and co-evolve with humans. The microecological composition of different systems is quite diverse and has its unique characteristics. The intestinal microecology is the largest and most studied microecological system in the human body (Wu et al., 2022). The number of its microbial communities can reach 1 × 10^14^, while the number of eukaryotic cells in the human body is around 1 × 10^13^, and about 1,500 kinds of bacteria are isolated (Vallianou and Tzortzatou-Stathopoulou, 2019). More microecological structures have been discovered with the rapid development of molecular biology techniques for microecological research. Under normal circumstances, microecology is in a state of balance in healthy organisms, and a balanced form between microorganisms and the host in different organisms is called microecological balance (Li et al., 2022). Microecology mainly studies the relationship and interaction between microbial communities and their hosts. In this review, we will discuss the correlations between microecology and human health, especially analyze the effects of microecological imbalance on human diseases, including lung cancer.

## Microecology and human health

### Functions of microecology

In addition to maintaining the balance of the internal and external environment, the microecological balance in the human body is also significant. Normal microecology is essential in promoting and preserving physiological functions, regulating nutritional metabolism, immune regulation, growth and development, and aging. Normal gut microbiota could adhere to and colonize the intestinal mucosa to form a bacterial membrane barrier and resist the invasion of foreign harmful bacteria. The gut microbiota can also synthesize essential vitamins and non-essential amino acids and promote the absorption of trace elements such as iron, magnesium, and zinc (Li et al., 2024). Gut microbiota can also produce bioactive metabolic substances that enter the blood circulation, and then affect the body’s metabolic process (Wang Y. et al., 2021; Debnath et al., 2021). In addition, the microbiota of gut and respiratory tract have a wide range of effects on the human immune system and immune function.

### Microecology and disease

In recent years, the relationship between microecological imbalances and human diseases has attracted much attention, the imbalances of microecology includes that between the microbiota and the organism, among different microorganisms, or in the unity of the microbiota, the host and the external environment (Li et al., 2022; Cai et al., 2022). With the rapid development of molecular biology technology, people have found more relationships between microecological imbalance and the occurrence and development of diseases, such as gut microbiota imbalance and inflammatory bowel disease, colorectal cancer, liver diseases and other digestive diseases (Jia et al., 2018; Albillos et al., 2020; Caruso et al., 2020). In addition, the relationships between respiratory microbiota imbalance and asthma, chronic obstructive pulmonary disease, interstitial lung disease, and lung cancer are also observed (Budden et al., 2019; Molyneaux et al., 2017; Lung Microbiota Promote Lung Cancer, 2019), which will open up a new path for the diagnosis and treatment of diseases.

A malignant tumor is one of the diseases that seriously threaten human health and the most common cause of human death. Although people have conducted a large number of studies on the pathogenesis and treatment of cancer, and the treatment methods of cancer are constantly developing, the incidence and mortality of cancer are still high (Global Burden of Disease Cancer C et al., 2022). It is urgent to reveal the new mechanism of tumor occurrence further to create new therapeutic measures to improve the prognosis of cancer patients. In addition to gene mutation, abnormal signal transduction, immune escape, and metabolic abnormalities, microecological imbalance may be another important mechanism to promote tumor development (Ge et al., 2021). Science published the findings of Nejman’s research team in 2020, which conducted a comprehensive analysis of 7 common tumor tissue microbiome through 16S ribosomal RNA (16S rRNA) sequencing, fluorescence *in situ* hybridization technology, transmission electron microscopy and tissue bacteria culture, including breast, lung, ovary, pancreas, melanoma, bone, and brain tumors (Nejman et al., 2020). This study found that each type of tumor is composed of unique intracellular bacteria and are present in both cancer and immune cells (Nejman et al., 2020). In recent years, there have been a lot of reports revealing the relationship between microecology and different cancers, including pancreatic cancer (Yu et al., 2021), breast cancer (Wang N. et al., 2021), colorectal cancer (Chen Q. F. et al., 2020), lung cancer (Bou Zerdan et al., 2022), and oral cancer (Chen et al., 2017).

### Development of research techniques in microecology

The development of microecology is closely related to the progress of related research techniques. With the development of molecular biology technology, the research methods of microecology have also developed from the traditional isolation and culture to the combined application of molecular biology detection and then to the application of metagenomics, transcriptomics, proteomics, metabolomics and other multi-omics technologies (Costa and Weese, 2019; Liu et al., 2023). Because it has been estimated that over 70% of human micobiota cannot be cultured, the traditional culture identification method has significant limitations in the study of microecology and it only partially reflects the composition and dynamic changes of the entire microbial community (Han et al., 2012; Vijayakumar et al., 2023). In recent years, with the wide application of rRNA small subunits and metagenomic sequencing technology, the research ability of microecology has significantly improved. At present, the two sequencing strategies widely used in the microbial field are bacterial 16S rRNA amplicon sequencing and metagenomic sequencing, both of them have the characteristics of high-throughput, fast, sensitive and accurate detection for qualitative and quantitative analysis of bacteria or strains (Wensel et al., 2022; Gao et al., 2021).

Bacterial 16S rRNA sequencing is a widely used gold standard for qualitative and quantitative analysis of bacteria. Bacterial rRNA can be divided into 5S, 16S and 23S according to sedimentation coefficient. Among them, the 16S rRNA gene sequence exists in all prokaryotes, and their sequences in general do not differ significantly owing to concerted evolution. Importantly, 16S rRNA gene has sufficient information which is suitable for the detection of molecular weight (Woo et al., 2008). Therefore, 16S rRNA sequencing has become an acceptable and commonly used means for identifying and quantitatively analyzing bacteria (Regueira-Iglesias et al., 2023). As we all know, 16S rRNA comprises conserved and variable regions, with the conserved regions reflecting the relationship among species and the variable regions reflecting the differences of species (Martinez-Porchas et al., 2017). The conserved regions can be used for general primer design, and the variable region can infer its classification (Comtet-Marre et al., 2023). Microbial composition and diversity were analyzed by high-throughput sequencing of the V3-V4 region of the 16S rRNA gene (Abellan-Schneyder et al., 2021). Almost all 16S rRNA gene sequences of prokaryotes have been successfully sequenced and preserved in public genome database. Therefore, for unknown bacteria, it is only necessary to amplify and sequencing the 16S rRNA gene and then conduct a comparative analysis of nucleotide sequences through public database to quickly determine the genus or species of the bacteria (Yarza et al., 2014). After the sequencing, taxon of different classification levels can be obtained through cluster analysis according to the similarity threshold of the 16S rRNA gene sequences, and the operational classification unit (OTU) can be obtained by annotation. Next, an OTU-based analysis first clusters sequences into different OTUs and then performs taxonomic assignment to understand the structure of microbiota, including information on abundance, diversity and differential characteristics (Gao et al., 2021).

Whole genome metagenomic sequencing is the process of “breaking” the whole genomic DNA into multiple fragments, directly sequencing these molecular fragments, and then analyzing the data after preprocessing (including sequencing quality control and “contaminated” sequence elimination) to understand the structure of the entire microbial community (Shi et al., 2022). The metagenomic data includes the whole genome information on functional composition, phylogenetic evolution, biodiversity, and differential characteristics of different microbial communities (Purushothaman et al., 2022). Metagenomic sequencing has the features of fast, accurate detection and complete coverage (Liu et al., 2021). In particular, metagenomic sequencing in bronchoalveolar lavage fluid significantly improved the efficiency of respiratory microecology research (Shi et al., 2020; Chen H. et al., 2020; Peng et al., 2021).

Although the development of sequencing technology has promoted the study of microecology to a large extent, the research on the interaction mechanism between microbiota and host cells still needs the data support of animal and cell experiments. Hence, the culture and purification of microbiota still play an irreplaceable role in relative studies (Lagier et al., 2018; Whelan et al., 2018).

## The relationship between microecology and lung cancer

Lung cancer ranks the second in the incidence of malignant tumors and the first in the death cause of malignant tumors (Aisner and Marshall, 2012). The prevention, diagnosis and treatment of lung cancer are always the focus of clinical attention. The etiology and pathogenesis of lung cancer are incredibly complex. Smoking is considered to be a significant risk factor for lung cancer, but <15% of patients with non-small cell lung cancer (NSCLC) smoke (Zhang et al., 2021), indicating that the occurrence of lung cancer is multifactorial. Chronic obstructive pulmonary disease and interstitial lung disease are associated with an increased risk of lung cancer (Terzikhan et al., 2016), indicating that inflammation plays a role in the pathogenesis of lung cancer. For example, lung cancer is often accompanied by pro-cancer inflammatory microenvironment (Tan et al., 2021). The literature has reported that microbiota dysregulation may affect cancer susceptibility in several ways, including modulation of host inflammatory responses, production of carcinogenic metabolites, genotoxicity and virulence effects, and cell cycle disruption (Mao et al., 2018). About 20% of cancers are closely associated with specific viral or bacterial infections (de Martel et al., 2020). In recent decades, several studies have reported that in addition to genetic and environmental factors, microbial imbalance plays an essential role in the occurrence and development of lung cancer (El Tekle and Garrett, 2023).

### Correlation between gut microbiota and lung cancer

Intestinal microbial community refers to microbiota inhabiting the human gastrointestinal tract, including bacteria, fungi, viruses, archaea, protists, etc. (Wu et al., 2023). Among all microbial ecosystems in the human body, the gastrointestinal tract accommodates the most significant number of microbiota (Garrett, 2015). The gut microbiota can be disrupted by malnutrition, overnutrition, inflammation, infectious diseases (mainly gastrointestinal) and medications (Kolodziejczyk et al., 2019; Shreiner et al., 2015). The scientific community also considers dysregulation of the gut microbiome as a potential marker of cancer.

The gastrointestinal and respiratory tracts are intrinsically linked and interact at multiple levels, known as the “gut-lung” axis (Dang and Marsland, 2019). Imbalance of gut microbiota can lead to respiratory and immune disorders, chronic inflammation, occurrence and development of lung cancer, and the response of respiratory diseases to treatment through the “gut-lung” axis (Enaud et al., 2020). The mechanisms related to gut microbiota and respiratory diseases mainly include (Budden et al., 2017): (1) Intestinal microbial components or metabolites such as lipopolysaccharides, short-chain fatty acids, and diamino-tyrosine enter the lungs through blood circulation and further affect lung tissue (Wang et al., 2023). (2) Gut microbiota lead to intestinal inflammation, and enterogenic cytokines/inflammatory mediators are transported to the respiratory system through the blood circulation to induce lung lesions (Chua et al., 2018). (3) Intestinal lymphocyte homing to the respiratory tract. Intestinal and respiratory mucosae have a commonality in genesis, physiological function, and organizational structure, such as access to the oropharynx, microvilli or cilia system, and IgA secretion. When stimulated by a pathogen, dendritic cells process the antigen and move to the lymph nodes, which in turn stimulate T and B lymphocytes to produce an immune response. It is worth noting that this mucosal lymphocyte homing is mucosal selective, adhesion molecules could select the areas and address elements. For example, gut microbiota can induce the production of type 2 and type 3 inherent lymphocytes, which could migrate to the respiratory tract through lymph and blood circulation to enhance the immune activity of the respiratory system (Routy et al., 2018). (4) The direct roles of microbiota. Studies have found that the composition of lung microbiota in mice with sepsis caused by abdominal infection is similar to that of gut microbiota (Dickson et al., 2016), suggesting that gut microbiota could directly migrate to the lungs. However, the mechanism of this migration remains unclear and needs further study.

Qian et al. (2022) performed 16S rRNA sequencing on the fecal samples of 55 patients with NSCLC and 15 healthy controls, and the results showed that the gut microbiota of NSCLC patients was significantly dysregulated. At the genus level, Prevotella, Gemmiger and Roseburia were significantly up-regulated in the NSCLC group. In addition, feces from NSCLC patients and healthy people were transplanted into the intestine of Lewis mice implanted with Lewis lung cancer cells, respectively. The results showed that tumors in mice transplanted with NSCLC feces grew significantly, and the levels of serum TNF-ɑ, IL-8, and other inflammatory factors were increased considerably. Furthermore, the extracted active *Prevotella copri* and inactivated *Prevotella copri* were transplanted into the intestinal tract of Lewis mice by gavage, respectively. The results also found that the tumors in the transplanted group with active *Prevotella copri* and the control group grew more rapidly than that in the transplanted group with inactivated *Prevotella copri*, and the inflammatory reaction in the lung and intestinal tissues was more prominent. These results indicated that intestinal *Prevotella copri* caused inflammatory response, immune dysregulation and lung cancer in mice (Qian et al., 2022). Qian et al. (2022) further performed 16S rRNA sequencing analysis on fecal samples from 61 lung cancer patients and 28 healthy controls. They divided lung cancer patients into three subgroups based on histopathology, including atypical adenomatous hyperplasia/adenocarcinoma *in situ* (AAH/AIS), minimally invasive adenocarcinoma (MIA), and invasive adenocarcinoma (IA). The results showed that the density and diversity of gut microbiota in lung cancer patients were decreased compared with healthy people, and different subgroups showed unique characteristics of pathogenic microbiota. Compared with the IA and MIA groups, the microbiota structure of the AAH/AIS and control groups was similar (Qin et al., 2022).

The relationship between microbiome and metabolome is also becoming one of the most active research areas in the biomedical field, including lung cancer. Short-chain fatty acids (SCFA) represent a beneficial metabolite in microbiota. A lot of evidences have shown that the gut microbiota and their specific metabolites are related to the pathogenesis of lung cancer, among which SCFA and bile acid are considered to be the key mediators (Blacher et al., 2017). Qian et al. (2022) also focused on the changes in metabolomics in their study of the relationship between NSCLC and gut microbiota. They found that lung cancer has a variety of different metabolites compared with healthy people, indicating that the pathogenesis of lung cancer may involve a wide range of metabolic disorders. The study results showed that the differential metabolites in serum samples were mainly related to aspartate, alanine, and glutamate metabolism. In the tissue samples, the prominent metabolites were primarily associated with GABAergic synapse, the intestinal immune network produced by IgA, and glutamatergic synapse (Qian et al., 2022). Gui et al. (2020) also reported that the bacteria containing butyric acid in the gut of NSCLC patients were significantly reduced, which might affect the progression and prognosis of lung cancer. These beneficial bacteria help to inhibit the growth of harmful bacteria in the gastrointestinal tract by improving intestinal barrier function, thus stabilizing intestinal homeostasis. These results suggested that structural changes in the gut microbiota are associated with different histopathological types of lung cancer. Compared with healthy people, the gut microbiota in lung cancer contains fewer bacteria that produce SCFA and have anti-inflammatory effects. In contrast, some pathogenic bacteria with pro-inflammatory or oncogenic effects are more abundant. Prevotella, Blastomyces and Rotella may be used as diagnostic, prognostic, or therapeutic targets in the diagnosis and treatment of lung cancer (Table 1).

**Table 1:** Relationship between gut/oral/lower respiratory tract microbiota and lung cancer.

Main findings	Variables	Sample type	References, Year
Prevotella, Gemmiger, and Roseburia upregulated	NSCLC and HC	Fecal sample	Qian et al. (2022)
Lower density and flora diversity	Lung cancer and HC	Fecal sample	Qin et al. (2022)
Butyrate-producing bacteria decreased	NSCLC and HC	Fecal sample	Gui et al. (2020)
Imbalanced *Fusobacterium nucleatum* and *Prevotella histicola*	Lung cancer and HC	Saliva sample	Jiang et al. (2022)
Lower abundance of Spirochaetia and Bacteroidetes, greater abundance of the Bacilli class and Lactobacillales order	Lung cancer and HC	Mouth rinse sample	Hosgood et al. (2021)
Higher Sphingomonas and Blastomonas, lower Acinetobacter and Streptococcus	Female lung cancer and HC	Saliva sample	Yang et al. (2018)
Lower airway dysbiotic signature was more prevalent in group IIIB-IV TNM stage, *Veillonella parvula*	Lung cancer with different stage and prognosis	Buccal and lower airway brushes	Tsay et al. (2021)
*Haemophilus influenzae*, *Streptococcus pneumoniae* and *Pseudomonas* spp.	Lung cancer	Bilateral protected specimen brush and lung tissue biopsy	Ioanas et al. (2002)
Intratumor bacteria are mostly intracellular, present in both cancer and immune cells	Different cancer types	Tumor and normal tissues	Nejman et al. (2020)
Firmicutes was predominant, Streptococcus and Veillonella were highly dominant in all isolated strains	Lung cancer and control	BALF and oral cavity	Sun et al. (2023)
Genus Streptococcus was significantly more abundant in cancer cases	Lung cancer and control	Protected bronchial specimen brushing	Liu et al. (2018)
higher abundance of orders Actinomycetales and Pseudomonadales were associated with worse DFS	Lung cancer	Tumor and distant normal lung samples	Peters et al. (2022)
The lower airways of patients with lung cancer were enriched for oral taxa (Streptococcus and Veillonella)	Lung cancer and control	Airway brushings	Tsay et al. (2018)

NSCLC, non-small cell lung cancer; HC, healthy control; BALF, bronchoalveolar lavage fluid; DFS, disease-free survival.

### Correlation between oral microbiota and lung cancer

The oral microbiota contains nearly 800 species and 20 million non-redundant genes, making it the second most abundant microbial community in the human body (Tierney et al., 2019), and it may enter deeper lower airways and lungs through continuous micro inhalation.

Segal et al. (2016) have shown that acellular bronchoalveolar lavage samples from half of the healthy people examined were enriched with oral taxa which were associated with local inflammatory responses characterized by elevated T-helper 17 (Th17) cells, and believed that this was the key regulating lung immune status in both health and disease. Jiang et al. (2022) conducted a macroproteomic analysis of unstimulated whole saliva, and the results showed that Actinobacteria and Firmicutes decreased in the lung cancer group, while the number of Fusobacteria and Proteobacteria increased. In addition, a large cohort study found that an increase in the abundance of Lactobacillus in the mouth was strongly associated with a risk of lung cancer (Hosgood et al., 2021).

In terms of specific populations, Yang et al. (2018) evaluated the saliva microbiome of 75 non-smoking female lung cancer patients and 172 matched healthy individuals by 16S rRNA sequencing, the results showed that the microbial diversity and richness were significantly decreased in lung cancer patients compared with the control. Non-smoking lung cancer patients had relatively high Sphingomonas and Blastomonas, while the control group had relatively high Acinetobacter and Streptococcus. In terms of mechanisms, Tsay et al. (2021) reported that in 83 lung cancer patients, the lower respiratory tract microbiome was similar to the oral microbiome, with rich Streptococcus, Prevotella and Veillonella genera associated with poorer survival in patients with stage I-IIIA NSCLC. This characteristic abundance of oral flora is associated with up-regulation of p53, PI3K/PTEN, ERK, and IL6/IL8 pathway transcription in the lower airway. Moreover, the authors suggest that NSCLC patients with stage IIIB-IV are more likely to have a rich oral symbiotic microbiota in the lower airway than patients with stage I-IIIA (Table 1).

### Correlation between lower respiratory tract microbiota and lung cancer

#### Overview of lower respiratory tract microecology

The respiratory tract communicates with the outside world. With cricoid cartilage as the boundary, the respiratory tract is divided into the upper and lower respiratory tract. Conventional culture methods cannot quickly cultivate the microbiota of the lower respiratory tract. It has always been believed that the roles of bacterial communities in the upper respiratory tract have in preventing respiratory pathogens from establishing an infection environment and spreading to the lower respiratory tract has accumulated (Man et al., 2017). With the development of microbial molecular biology technology, more and more studies have proved that microbiota exist in the lower respiratory tract. These microbiomes, their metabolites and the surrounding environment constitute the microecology of the lower respiratory tract (Lanaspa et al., 2017). In 2014, Dickson et al. (2014) proposed an island model, suggesting that respiratory microecology is a process of gradient change from the nosehole to the lungs. From the upper respiratory tract to the lower respiratory tract, the diversity and abundance of microbiota gradually decrease. The nostrils are mainly composed of Firmicutes and Actinomyces, while Firmicutes, Proteobacteria, and Bacteroidetes exist in the oropharynx (Lemon et al., 2010). The nasal microbiota is closer to the skin microbiota and contributes little to the lung microecology. Other studies have shown that the lung microbiota of normal adults is similar to that of the oropharynx, in which the common bacteria mainly include Firmicutes, Proteobacteria and Bacteroides at the phylum level, and Prevotella, Veillonella, Streptococcus, and Pseudomonas at the genus level (Dickson et al., 2016). At present, the source of the lung microbiome is not very clear, and air people breathe in also contains a certain amount of bacteria. Some studies have speculated that the lung microbiota may be alien microorganisms that enter the lung through aspiration or air inhalation, and the formation of lung specific microbiota results from the joint action of environmental exposure factors and host defense response (Dong et al., 2021). Studies have shown that the microecological imbalance of lower respiratory tract is related to various lung diseases, such as asthma, chronic obstructive pulmonary disease, pulmonary interstitial fibrosis and lung cancer (Budden et al., 2019; Man et al., 2017; Lanaspa et al., 2017).

#### Relationship between lower respiratory tract microecological imbalance and lung cancer

Compared with the study of intestinal microbiota, the correlation between lower respiratory tract microbiota and lung cancer is still in the preliminary stage. As early as 2002, Ioanas et al. (2002) analyzed lung tissue biopsies or bilateral protective cell brush samples from 41 operable lung cancer patients by quantitative bacterial culture. The study found that 17 of 41 patients (41%) had more than one potentially pathogenic microbe colonized in their bronchus. The most common isolates were *Haemophilus influenzae* (35%), *Streptococcus pneumoniae* (13%) and *Pseudomonas* spp. Of course, the culture method dramatically limited the number of microorganisms that can be found.

With the application of high-throughput sequencings such as 16S rRNA sequencing, a large number of lower airway or lung microbiota have been discovered, and the types of specimens available have gradually diversified, including bronchoalveolar lavage fluid, lung tissue, and bronchial brush samples, etc. (Nejman et al., 2020; Sun et al., 2023; Liu et al., 2018), which result in significant differences and even contradictory results among the data. This may be related to the combination of underlying diseases and antimicrobial use in the enrolled population, which suggests that we need to be cautious in interpreting relevant data, and this needs to be confirmed by further large prospective cohort studies.

Peters et al. (2022) sequenced 16S rRNA of microbiota in tumor and distant normal lung tissue samples from 46 patients with stage II NSCLC. The results showed that Bacteroides and Clostridia were found at the class level in normal lung tissue. At the order level, a high abundance of Bacteroidales and Clostridiales was associated with poor relapse-free survival (RFS), while high abundance in Burkholderiales and Neisseriales was associated with better RFS. When Tsay et al. (2018) analyzed the lower airway protective brush samples from 39 cases of lung cancer, 36 cases of benign lung diseases, and 10 healthy controls, it was found that lung cancer patients had more Veillonella and Streptococcus than those with benign lung diseases and healthy controls. Tsay et al. (2021) expanded the study cohort to enroll 148 subjects with pulmonary nodules, they finally included 83 lung cancer patients and obtained their lower airway protective brush samples, oral mucosa (representing the upper respiratory tract), and bronchoscopic background control samples. The results showed that the variety of lower respiratory tract samples was higher than that of upper respiratory tract and background control samples based on the Shannon index. There were also compositional differences between small cell lung cancer and NSCLC in lower airway samples (Table 1).

### Effects of microbiota dysbiosis on chemotherapy efficacy of lung cancer

Studies have shown that microbiota can influence the effect of cytotoxic drug therapy by regulating metabolism or immune response. Cyclophosphamide-induced immunogenicity bacteria such as Enterococcushirae and Barnesiellaintestinihominis through the intestinal wall transferred to lymphoid organs, respectively, which stimulates the production of antitumor T lymphocytes and release of IFN-gamma delta T cells increased (Viaud et al., 2013; Daillère et al., 2016). Zhao et al. (2020) found a response to chemotherapy in lung cancer patients correlated with increasing intestinal acid bacillus, streptococcus mutans, *Enterococcus casseliflavus*, and Granulicella levels; and intestinal fermentation streptococcus, *Megasphaera micronuciformis* and inert coli abundance were higher in non-responders. On the other hand, chemotherapy might cause microecological dysregulation, resulting in a decrease in the total number of gut microbiota and changes in the composition of gut microbiota, leading to a higher incidence of adverse events, including increased Clostridioides and Candida infections (Toi et al., 2021; Seelbinder et al., 2023; Chudzik-Rząd et al., 2022).

Remarkably, gut microbiota has been also shown to be associated with cancer-related fatigue (CRF) in advanced lung cancer patients receiving first-line chemotherapy, with an increase in pro-inflammatory microbiota in patients with severe CRF, such as Escherichia-Shigella genus and Enterobacteriaceae family; However, patients with mild CRF have increased anti-inflammatory microbiota, such as Lachnospiraceae and Lachnospiraceae (Wei H. et al., 2022) (Table 2).

**Table 2:** Relationship between microbiota and the treatment of lung cancer.

Main findings	Treatment	Microbiota origin	References
Bacteria stimulated the generation of T helper 17 cells and memory Th1 immune responses	Cyclophosphamide	Intestinal microbiota	Viaud et al. (2013)
*Enterococcus hirae* and *Barnesiella intestinihominis* involved	Cyclophosphamide	Intestinal microbiota	Daillère et al. (2016)
Specific gut microbiome were associated with clinical outcomes of chemotherapy	Chemotherapy	Intestinal microbiota	Zhao et al. (2020)
Incidence of *Clostridium difficile* infection was significantly higher in patients treated with paclitaxel	Chemotherapy	Intestinal microbiota	Toi et al. (2021)
High Candida levels in cancer patients’ stool correlated with greater metabolically flexibility but less robust bacterial communities	Chemotherapy, immunotherapy	Intestinal microbiota	Seelbinder et al. (2023)
Increasing proinflammation taxa in severe CRF patients and anti-inflammation taxa growing in mild CRF patients	Chemotherapy	Intestinal microbiota	Wei H. et al. (2022)
*Alistipes putredinis*, *Bifidobacterium longum*, and *Prevotella copri* were enriched in responder	Immunotherapy	Intestinal microbiota	Jin et al. (2019)
*Veillonella dispar* was dominant in the high-PD-L1 group; Neisseria was higher in the low-PD-L1 group	Immunotherapy	Respiratory tract microbiota	Jang et al. (2021)
The presence of specific bacterial DNA in blood at M0 was associated with response and clinical benefit	Immunotherapy	Blood microbiota	Ouaknine Krief et al. (2019)
pATB therapy but not cATB therapy is associated with a worse treatment response and OS	Antibiotic therapy, immunotherapy	Intestinal microbiota	Pinato et al. (2019)
The negative influence of antibiotic use	Antibiotic therapy, immunotherapy	Intestinal microbiota	Hakozaki et al. (2019)
Fecal microbiota transplantation from cancer patients who responded to ICIs into germ-free or antibiotic-treated mice ameliorated the antitumor effects of PD-1 blockade	Immunotherapy	Intestinal microbiota	Routy et al. (2018)

CRF, cancer-related fatigue; pATB, prior antibiotic therapy; cATB, concurrently antibiotic therapy; ICIs, immune checkpoint inhibitors.

### Effects of microbiota dysbiosis on immunotherapy efficacy of lung cancer

In recent studies, the microbiome is a crucial modulator of the immune response against cancer cells, affecting immunotherapy’s efficacy. Jin et al. (2019) included 37 patients with advanced NSCLC treated with nivolumab—assessment of gut microbiota profile by 16S rRNA sequencing. The results showed that patients who responded to immunotherapy had a higher gut microbiome diversity at the beginning of treatment. Its composition was stable during treatment, and patients with a high microbiome diversity had significantly longer progression-free survival than those with a low microbiome diversity. Differential analysis showed that *Alistipes putredinis*, *Bifidobacterium longum*, and *Prevotella copri* were highly enriched in the response group. Research of systemic immune responses showed that patients with a rich gut microbiome diversity have higher peripheral content of unique memory CD8 + T cells and natural killer cell subsets upon anti-PD-1 treatment (Jin et al., 2019). Another cohort study involving 60 patients with advanced NSCLC and 40 patients with renal cell carcinoma who received anti-PD-1 immunotherapy found that the efficacy of immunotherapy was related to the composition of the gut microbiota. The abundance of *Akkermansia muciniphila* in the feces of patients who respond to immunotherapy is significantly higher than that of non-responders, and oral administration of *Akkermansia muciniphila* could re-establish T cell immunity against tumors in non-responsive patients (Routy et al., 2018). Jang et al. (2021) analyzed the relationship between lung microbiota and lung cancer immunotherapy. In this study, 16S rRNA sequencing of bronchoalveolar lavage fluid was performed in 84 lung cancer patients. The results showed that there were no significant differences in *α* diversity and *β* diversity between groups with low (< 10%) and high (> 10%) expression of PD-L1. However, Veillonelladispa was dominant in the group with high PD-L1 expression, and the number of Neisseria in the low PD-L1 expression group was significantly higher. In the immunotherapy response group, V. DAPPAR predominated, while in the immunotherapy non-response group, Haemophilusinfluenzae and Neisseriaperflava predominated. The results suggested that the abundance of Neisseria and V. DAPPAR had significantly different effects on the expression level of PD-L1 and the response to immunotherapy. Ouaknine Krief et al. (2019) also analyzed the influence of blood microbiome on the efficacy of lung cancer immunotherapy, and the results showed that the presence of specific bacterial DNA in the blood was closely related to the effectiveness of immunotherapy in NSCLC patients. Peptostreptococcae, Paludibaculum and Lewinella were associated with clinical benefit, while Gemmatimonadaceae was associated with tumor progression after immunotherapy.

Recently, several studies have shown that lung cancer patients treated with antibiotics before immunotherapy have significantly worse responses to immunotherapy and survival (PFS and overall survival) than those who do not receive antibiotics, which might be related to the imbalance of gut microbiota caused by antibiotics (Pinato et al., 2019; Hakozaki et al., 2019). Interestingly, there were no such differences in patients treated with antibiotics and immunotherapy simultaneously. Routy’s study showed that after the use of antibiotics, intestinal Akkermansiamuciniphila bacteria decreased, leading to a reduction in the effect of immunotherapy (Routy et al., 2018). Further analysis shows that the response of fecal microbiota transplantation (FMT) patients could rebuild no answer to the immunotherapy (Routy et al., 2018) (Table 2).

### Possible mechanisms by which dysregulation of microbiota promotes the occurrence and development of lung cancer

**Figure 1:**
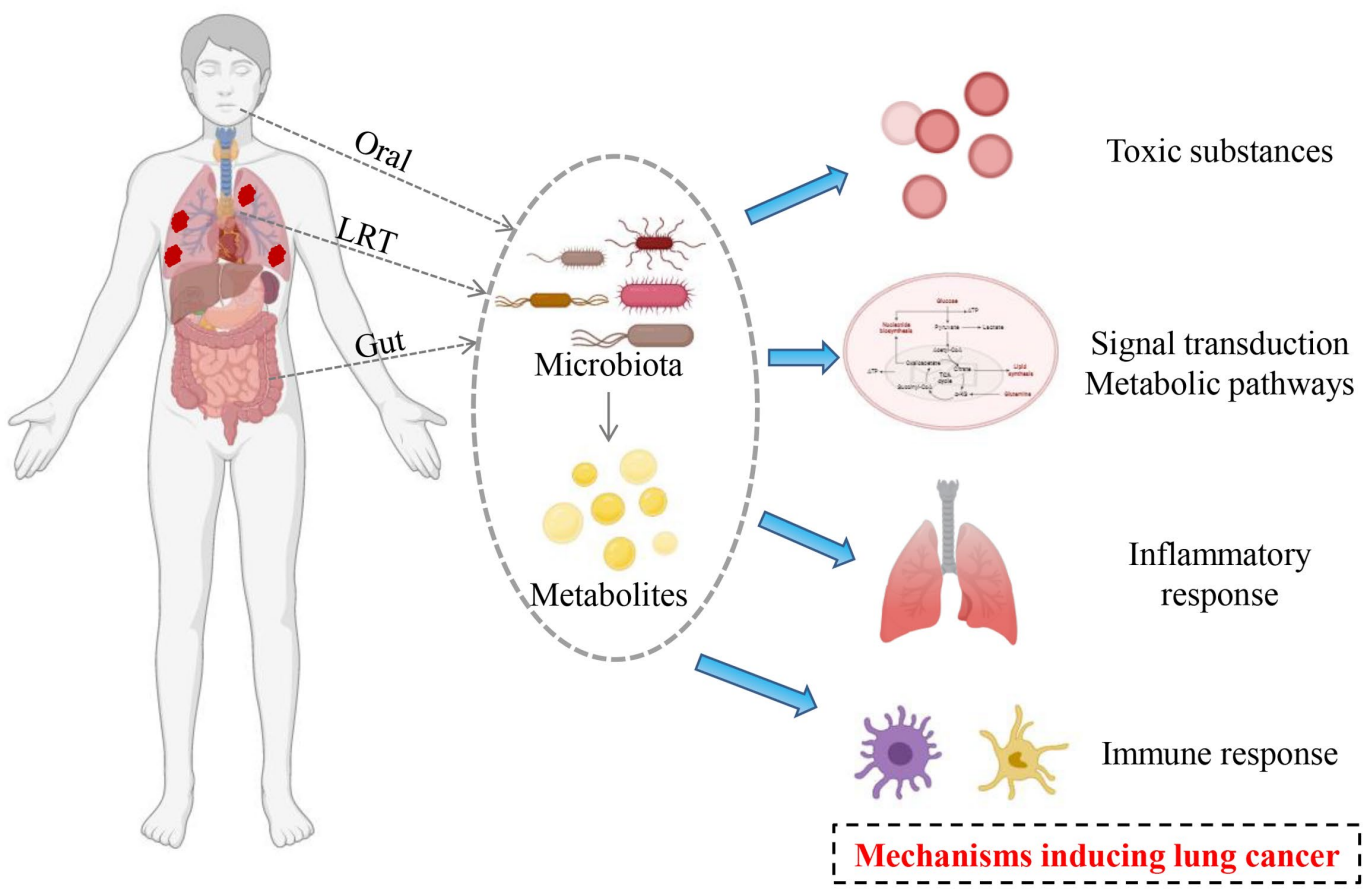
Potential mechanisms mediating by microbiota in the occurrence and development of lung cancer.

Microbial microecological imbalance can cause the occurrence and development of lung cancer by producing excessive toxic substances and mediating the inflammatory response (Figure 1).

#### Production of toxic substances

Many microorganisms have been shown to produce compounds that can cause DNA damage, cell cycle arrests, and genetic material instability, such as *Escherichia coli* and other Enterobacteriaceae bacteria code for polydactyl synthase, which produces Colibactin. The latter can lead to tissue gene mutation and DNA damage, leading to the occurrence and development of tumors (Oliero et al., 2022; Pleguezuelos-Manzano et al., 2020). Endotoxins, proteases, fibrinolytic enzymes, and fatty acids in the microorganisms themselves and their metabolites have toxic effects on host cells, which could directly induce cell carcinogenesis and promote the occurrence of tumors. Microbial infection can escape from the immune system to form virulence factors such as oxygen free radicals, nitric oxide, and matrix metalloproteinases to promote the occurrence of tumors.

#### Regulate signal transduction and metabolic pathways

Microbiota disorders could promote the occurrence of tumors by changing the cell signal transduction pathways. Microbiota could be triggered by mitogen-activated protein kinase (MAPK) ways to induce cell proliferation, increase the genetic mutations, and improve tumor metastasis rate and the incidence of a disease (Chen et al., 2021). At the same time, the bacteria infection caused by the intracellular accumulation effect can be achieved by adjusting B-lymphoma-2 (Bcl-2) family protein expression or inactivated retinoblastoma inhibitory protein to inhibit apoptosis and promote the transformation of cancer cells (Cui et al., 2018). The lower airways of patients with lung cancer were enriched for oral microbiota (such as Streptococcus and Veillonella), which was associated with up-regulation of the ERK and PI3K signaling pathways. Importantly, the airway epithelial cells were *in vitro* exposed to Veillonella, Prevotella, and Streptococcus, which could lead to up-regulation of these same signaling pathways (Tsay et al., 2018).

Studies have found that some metabolites, such as choline phosphate, taurine, glutathione, glutamine and arginine, are significantly altered in lung cancer. Microbial metabolism of bile acids and proteins could lead to formaromatic amines and sulfides to promote tumor growth. Hosgood et al. (2021) studied non-smoking female lung cancer patients and compared their sputum microbiota with those of ordinary people in the area. It was found that the relative abundance of Streptococcus granulosus (6.1%), trophic deficiency bacteria (1.5%), and Streptococcus (40.1%) in the sputum of lung cancer patients was significantly higher than that of ordinary people (2, 0.085 and 19.8%, respectively). It was speculated that respiratory microorganisms might affect the metabolism of carcinogens such as polycyclic aromatic hydrocarbons *in vivo*. It has been confirmed that Streptococcus granulosus could affect the development of lung cancer by participating in the metabolic pathway of putputylamine and polyamine, and the degradation of putputylamine and polyamine can affect the cell cycle. Ye et al. (2016) conducted primary cell culture on surgically resected lung cancer tissues and found that the toll-like receptor (TLR) 4 and TLR9 pathways were activated after culture stimulated by Gram-negative bacilli, resulting in increased lipid synthesis, leading to tumor progression and metastasis.

#### Inflammatory response

Inflammatory responses can contribute to cancer development by inducing genomic mutations, abnormal tissue repair, and proliferative reactions. Microbiota can induce chronic infection or produce cytotoxins, promote host inflammatory response, cause DNA damage and cell cycle abnormalities, and promote tumor development and metastasis. Infiltrating neutrophils also promotes tumor-associated inflammation, angiogenesis, and metastasis. Brenner et al. found that pulmonary *M. tuberculosis* infection increased the risk of lung cancer (Myer et al., 2011). Jungnickel et al. (2017) found that the tail vein injection of lung cancer cells successively in mice exposed to the inseparable type of *Haemophilus influenzae* (Nontypeable Haemophilus influenza, NTHi), mice exposed to NTHi group of pulmonary metastasis nodule number and volume are increased in the unexposed group increase, Further studies showed that NTHi up-regulates the expression of IL-17C through toll-like receptors 2/4, and IL-17C induced the infiltration of neutrophils in tumor microenvironment and promotes tumor development. Basic experiments have confirmed that *Streptococcus pneumoniae* could up-regulate the expression of interleukin-6 through toll-like receptor 2, thus promoting lung cancer cell metastasis (Gowing et al., 2017). Another study found that increased WeiRong aureus inflammation factors were associated with releasing further increased regulation protein kinase-phosphatidyl inositol three kinase, which further affect the development of lung cancer (Tsay et al., 2018).

#### Interference with immune response

Studies have shown that lung microecology is related to the lung’s immune tolerance to malignant tumor metastasis (Le Noci et al., 2018), and antibiotics or probiotics can promote the immune response to tumor lung metastasis. Mice fogging with vancomycin reduced the number of regulatory T cells while increasing the activity of T cells and NK cells, significantly decreasing tumor metastasis. Probiotics could also inhibit lung metastasis of malignant tumors by promoting the body’s immune response. It is now known that both the microbiota’s cell wall components and metabolites regulate the host’s immune response to microbial and environmental stimuli and further affect the body’s immune cells through various mechanisms. Liang et al. (2016) showed that immune cells such as T regulatory cells, M2 macrophages and activated neutrophils promoted tumor growth in the lung microenvironment. Gur et al. (2015) found through *in vitro* cell experiments that *C. nucleatum* produces a particular protein, Fap2, which binds to TIGIT (an inhibitory receptor) on the surface of T cells and natural killer cells, thereby blocking the cytotoxic effects of immune cells on tumor cells. The surface polysaccharide derived from *Bifidobacterium longum* can inhibit the lung-selective Th17 response (Schiavi et al., 2016). Song further demonstrated that microbially induced Th17 can promote lung cancer cell proliferation and angiogenesis (Song et al., 2019). Tsay proved that the microbiome can regulate immune response to cancer, mouse model produced enterotoxin fragile Bacteroides in Th17 and triggered signal conduction activation of transcription factor 3 (signal transducer and activator of transcription 3, STAT3). Cancer could be induced by a Th17-dependent pathway (Tsay et al., 2021). Another experiment showed that mice exposed to Haemophilus parainfluenza for 6 months increased the number of lung inflammatory cells and promoted the occurrence of lung tumors by promoting micro angiogenesis and up-regulating the expression of hypoxia-induciblefactor1 (Eckle et al., 2014). Tsay et al. (2018) found that the abundance of Streptococcus and Veyonea in the airways of lung cancer patients was significantly up-regulated, which was related to the up-regulation of PI3K and ERK, and the up-regulation of PI3K pathway was considered to be an early event in the occurrence of lung cancer. Jin et al. (2019) have shown that the increase of lung bacteria in lung cancer model mice led to the proliferation of local lung γδT cells and the secretion of pro-inflammatory factors IL-17 and IL-23, thus promoted the growth of lung cancer. The γδT cells encouraged the infiltration of neutrophils and release of pro-inflammatory factors, further promoted tumor development. However, the tumor volume in mice was significantly reduced after anti-infective treatment.

## Conclusion

In summary, human microecology, including intestinal microbiota, oral microbiota, lower airway and lung microbiota, regulates multiple hosts’ physiological and pathological processes, including metabolism, inflammation, and immune regulation. Microecological imbalance plays an important role in the occurrence and development of lung cancer and the efficacy of chemotherapy or immunotherapy. Although the current research still has many limitations, such as the diversity of samples tested and the inconsistency of results caused by the lack of uniform standards and lack of prospective cohort studies based on large populations. Importantly, the existing research results are encouraging and providing strong evidences for the role of microbiome dysregulation in lung cancer, laying the foundation for the design of microbiome-centered detection and intervention. This field will provide a new strategy to improve the early diagnosis of lung cancer by detecting the pernicious bacteria in gut, oral cavity and lower respiratory tract. Moreover, it will provide new targets and improve the treatment method for the prevention of lung cancer, such as the combination of anti-microbial treatment and chemotherapy or immunotherapy, the design of probiotics, and the development of bacteria-derived molecular medicine.
